# Aldehyde Dehydrogenase 2 Activator Augments the Beneficial Effects of Empagliflozin in Mice with Diabetes-Associated HFpEF

**DOI:** 10.3390/ijms231810439

**Published:** 2022-09-09

**Authors:** Guodong Pan, Bipradas Roy, Shailendra Giri, David E. Lanfear, Rajarajan A. Thandavarayan, Ashrith Guha, Pablo A. Ortiz, Suresh Selvaraj Palaniyandi

**Affiliations:** 1 Division of Hypertension and Vascular Research, Department of Internal Medicine, Henry Ford Health System, Detroit, MI 48202, USA; 2 Department of Physiology, Wayne State University, Detroit, MI 48202, USA; 3 Department of Neurology, Henry Ford Health System, Detroit, MI 48202, USA; 4 Heart and Vascular Institute, Henry Ford Hospital, Detroit, MI 48202, USA; 5 Center for Health Policy and Health Services Research, Henry Ford Hospital, Detroit, MI 48202, USA; 6 Department of Cardiovascular Sciences, Houston Methodist Research Institute, Houston, TX 77030, USA

**Keywords:** HFpEF, diabetes, heart failure, 4-hydroxy-2-nonenal, aldehyde dehydrogenase 2, empagliflozin, Alda-1, cardiac function, ALDH2*2 mutant mice

## Abstract

To ameliorate diabetes mellitus-associated heart failure with preserved ejection fraction (HFpEF), we plan to lower diabetes-mediated oxidative stress-induced 4-hydroxy-2-nonenal (4HNE) accumulation by pharmacological agents that either decrease 4HNE generation or increase its detoxification.A cellular reactive carbonyl species (RCS), 4HNE, was significantly increased in diabetic hearts due to a diabetes-induced decrease in 4HNE detoxification by aldehyde dehydrogenase (ALDH) 2, a cardiac mitochondrial enzyme that metabolizes 4HNE. Therefore, hyperglycemia-induced 4HNE is critical for diabetes-mediated cardiotoxicity and we hypothesize that lowering 4HNE ameliorates diabetes-associated HFpEF. We fed a high-fat diet to ALDH2*2 mice, which have intrinsically low ALDH2 activity, to induce type-2 diabetes. After 4 months of diabetes, the mice exhibited features of HFpEF along with increased 4HNE adducts, and we treated them with vehicle, empagliflozin (EMP) (3 mg/kg/d) to reduce 4HNE and Alda-1 (10 mg/kg/d), and ALDH2 activator to enhance ALDH2 activity as well as a combination of EMP + Alda-1 (E + A), via subcutaneous osmotic pumps. After 2 months of treatments, cardiac function was assessed by conscious echocardiography before and after exercise stress. EMP + Alda-1 improved exercise tolerance, diastolic and systolic function, 4HNE detoxification and cardiac liver kinase B1 (LKB1)-AMP-activated protein kinase (AMPK) pathways in ALDH2*2 mice with diabetes-associated HFpEF. This combination was even more effective than EMP alone. Our data indicate that ALDH2 activation along with the treatment of hypoglycemic agents may be a salient strategy to alleviate diabetes-associated HFpEF.

## 1. Introduction

Multiple factors contribute to heart failure with preserved ejection fraction (HFpEF), a condition that remains poorly understood and difficult to study or treat. The protean nature of HFpEF makes it unsurprising that there is not a single animal model that can mimic all the characteristics of HFpEF [1]. This can be cleverly segregated into individual models of HFpEF based on their etiopathophysiology, such as models of atrial fibrillation-mediated HFpEF, pulmonary hypertension-mediated HFpEF and diabetes-mediated HFpEF [1]. In this study, our focus will be on HFpEF associated with type-2 diabetes mellitus (type 2 DM). 

Among various factors in diabetes, hyperglycemia-mediated oxidative stress has been regarded as critical for end-organ damage via multiple pathways [2,3]. Oxidative stress is a complex and continuous process. Reactive oxygen species (ROS) generation is the initiating event of oxidative stress. Although ROS have a short half-life, they produce 4-hydroxy-2-nonenal (4HNE) similar to reactive carbonyls by lipid peroxidation. In addition, 4HNE is known to form adducts with proteins, DNA and extracellular matrix, and we have shown increased 4HNE adducts in both type-1 and type-2 diabetic hearts compared to controls [4,5,6], which contribute to cardiac damage and dysfunction [7]. In myocardial cells, 4HNE is detoxified by glutathione, aldehyde dehydrogenases (ALDHs) and aldose reductase [8]. We showed a significant increase in 4HNE adducts in cultured cardiac cells [9,10,11,12] and cardiac tissue [4,5,6,13], which is augmented by reduced ALDH2 activity by employing either diabetic insult or pharmacological inhibition or genetic mutation. 

A single point E487K mutation in ALDH2 (prevalent in East Asians) known as ALDH2*2 reduces its activity to ~3% in homozygotes (ALDH2*2/2) and ~30–50% in heterozygotes (ALDH2*2/1). We employed ALDH2*2 knock-in heterozygous mutant mice, which mimic East Asians with the ALDH2*2 mutation that have intrinsically low ALDH2 activity. In fact, a recent clinical study in East Asians with the ALDH2*2 mutation demonstrated an association between ALDH2*2 and HFpEF in patients who have diabetes and other comorbidities [14]. Even before this study, we were the first group to demonstrate that ALDH2*2 mutant mice with type-2 DM exhibited an exacerbation of HFpEF features relative to wild-type mice with type-2 DM [6]. 

In this study, we plan to only use pharmacological approaches as the majority of current therapeutic management is still implemented through drugs. In our earlier study, we decreased cardiac 4HNE adduct levels in ALDH2*2 diabetic mice by decreasing hyperglycemia via empagliflozin (EMP), a sodium-glucose cotransporter (SGLT) 2 inhibitor, and thereby improved cardiac function via altering metabolic signaling [15]. Therefore, in addition to decreasing 4HNE generation via EMP, we also want to increase 4HNE detoxification via ALDH2 activation by Alda-1, a small molecule ALDH2 activator. Alda-1 was first shown to increase both wild-type (WT) ALDH2 and ALDH2*2 activity and decrease 4HNE adductions in vitro and in the myocardium of rats subjected to ischemia-reperfusion [16]. 

Therefore, in this study, we will test the hypothesis that combining both lowering cardiac 4HNE adducts by reducing hyperglycemia-mediated oxidative stress and increasing 4HNE detoxification by ALDH2 activation should ameliorate DM-associated HFpEF in ALDH2*2 mice. 

## 2. Results

### 2.1. Effects of EMP, Alda-1 and EMP + Alda-1 on HFD-Induced Body Weight (BW) Gain and Type-2 Diabetes-Mediated Hyperglycemia 

**Figure 1:**
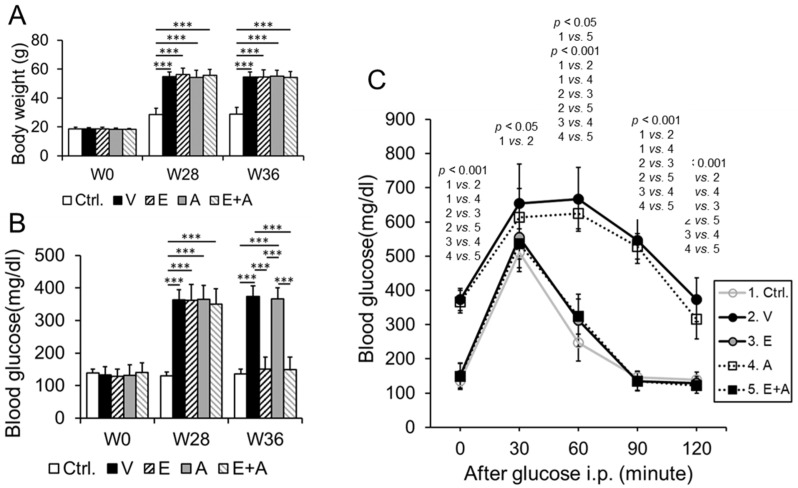
Changes in body weight gain, hyperglycemia and glucose intolerance in diabetic ALDH2*2 mutant mice. Body weight (**A**), blood glucose (**B**) and blood glucose tolerance (**C**) data were shown from non-diabetic control ALDH2*2 mutant mice (Ctrl.), vehicle-treated ALDH2*2 mutant diabetic mice (V), EMP-treated ALDH2*2 mutant diabetic mice (E), Alda-1-treated ALDH2*2 mutant diabetic mice (A) and EMP + Alda-1-treated ALDH2*2 mutant diabetic mice (E + A). Data are presented as mean ± standard error of the mean (SEM). n = 8 of each group. Weeks 0, 28 and 36 were denoted as W0, W28 and W36, respectively. In Figure 1A, *** *p* < 0.001 vs. Ctrl.; In (**B**), *** *p* < 0.001 vs. Ctrl., E and A.

HFD feeding increased body weight gain in ALDH2*2 mice compared to chow fed ALDH2*2 mice (Figure 1A). This increase in body weight gain was not reduced by vehicle, EMP, Alda-1 and EMP + Alda-1 treatments (Figure 1A). As expected, HFD-induced type-2 diabetes in ALDH2*2 mice increased hyperglycemia compared to chow-fed ALDH2*2 mice (Figure 1B). Among the treated groups, EMP and a combination of EMP and Alda-1 treatments decreased type-2 diabetes-mediated hyperglycemia compared to the vehicle (Figure 1B). However, Alda-1 treatment did not reduce hyperglycemia (Figure 1B). 

### 2.2. Effects of EMP, Alda-1 and EMP + Alda-1 on HFD-Induced Type-2 Diabetes Mediated Glucose Intolerance 

Similar to hyperglycemia, we found increased glucose intolerance in vehicle-treated type-2 diabetic mice compared to chow-fed control mice (Figure 1C). EMP alone and as well as in combination with Alda-1 significantly improved the glucose tolerance compared to vehicle and Alda-1 (Figure 1C). 

### 2.3. Effects of EMP, Alda-1 and EMP + Alda-1 on Improving Cardiac Function in Conscious Echocardiography 

**Figure 2:**
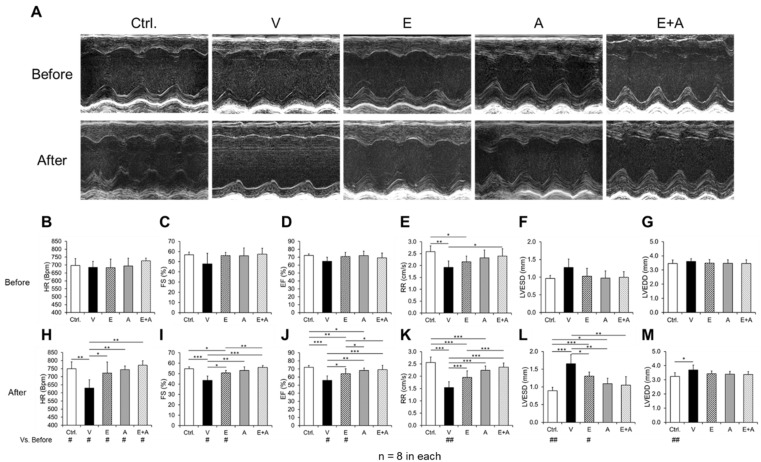
Changes in cardiac functional indices in diabetic ALDH2*2 mutant mice exhibiting HFpEF with vehicle, EMP, Alda-1 and EMP + Alda-1 treatments. The representative ultrasound images of our experimental groups were shown (**A**). Cardiac functional parameters measured before exercise stress: heart rate (HR) (**B**), % fractional shortening (FS) (**C**), % ejection fraction (EF) (**D**), relaxation rate (RR) data (**E**), left ventricular end-systolic diameter (LVESD) (**F**) and left ventricular end-diastolic diameter (LVEDD) (**G**) were shown from non-diabetic control ALDH2*2 mutant mice (Ctrl.), vehicle-treated ALDH2*2 mutant diabetic mice (V), EMP-treated ALDH2*2 mutant diabetic mice (E), Alda-1 treated ALDH2*2 mutant diabetic mice (A) and EMP and Alda-1 combinedly treated ALDH2*2 mutant diabetic mice (E + A). The cardiac functional parameters changes after exercise stress were shown as below: HR (**H**), % FS (**I**), % EF (**J**), and RR (**K**), LVESD (**L**) and LVEDD (**M**). Data are presented as mean ± standard error of the mean (SEM). n = 8 per group. * *p* < 0.05, ** *p* < 0.01 and *** *p* < 0.001 refer to the difference between individual groups as shown in before and after exercise stress. ^#^ *p* < 0.05 and ^##^ *p* < 0.01 vs. before exercise stress of that particular group.

We presented echocardiography images (Figure 2A) and cardiac functional parameters before (Figure 2B–G) and after (Figure 2H–M) exercise stress. There was no difference in heart rates (Figure 2B), the systolic functional parameters, i.e., % FS (Figure 2C) and % EF (Figure 2D) before exercise stress in vehicle-treated mice compared to control mice. There was a significant decrease in RR, diastolic functional parameter (Figure 2E), an increased left ventricular end-systolic dimension (LVESD, Figure 2F), but no statistics difference after analysis. There was no difference in left ventricular end-diastolic dimension (LVEDD, Figure 2G). Treatments with EMP + Alda-1 attenuated diabetes-induced decreases in the RR (Figure 2E). 

After exercise stress, the heart rate was significantly increased in control mice and decreased in vehicle-treated mice compared to before exercise stress (Figure 2H). Treatments with EMP, Alda-1 and EMP + Alda-1 combination increased heart rate after exercise stress (Figure 2H). There was no difference in % FS (Figure 2I) and % EF (Figure 2J) in control mice after exercise stress, however, the % FS (Figure 2I) and % EF (Figure 2J) were significantly decreased after exercise stress with vehicle treatment. Diabetes-mediated decreases in % FS and % EF were attenuated by EMP, Alda-1 and EMP + Alda-1 combination treatments (Figure 2I,J). Most importantly, the EMP + Alda-1 combination treatment improved the % FS and % EF even better than the individual treatment of EMP (Figure 2I,J). Strikingly, the RR was reduced (Figure 2K) in vehicle treatment compared to controls, which was further improved significantly by treatments with EMP, Alda-1 and EMP + Alda-1 combination. Most importantly, EMP + Alda-1 combination treatment improved the RR better than EMP alone (Figure 2K). The LVESD and LVEDD did not change significantly before exercise stress (Figure 2F,G). However, after the exercise stress, the LVESD was significantly increased in the vehicle-treated group, which was significantly reduced with EMP, Alda-1 and the EMP + Alda-1 combination (Figure 2L). On the other hand, EMP, Alda-1 and EMP + Alda-1 combination treatments did not decrease LVEDD, which was higher in the vehicle treatment (Figure 2M). 

### 2.4. Effects of EMP, Alda-1 and EMP + Alda-1 on Exercise Tolerance 

**Figure 3:**
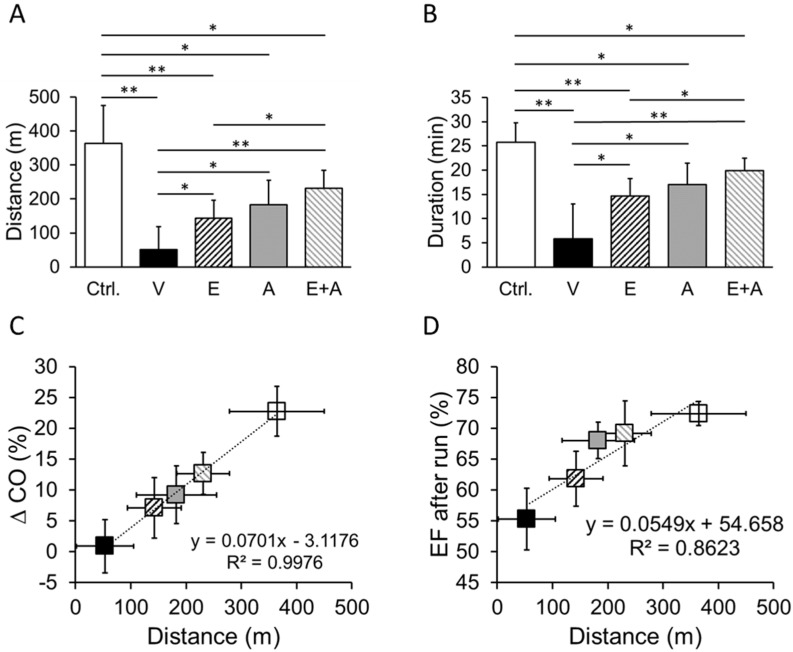
Changes in the running distance with treadmill exercise in diabetic ALDH2*2 mutant mice exhibiting HFpEF with vehicle, EMP, Alda-1 and EMP + Alda-1 treatments. Running distance (**A**), running duration (**B**), correlation between by cardiac output with distance (**C**) and ejection fraction with distance (**D**) were shown from non-diabetic control ALDH2*2 mutant mice (Ctrl.), vehicle-treated ALDH2*2 mutant diabetic mice (V), EMP-treated ALDH2*2 mutant diabetic mice (E), Alda-1 treated ALDH2*2 mutant diabetic mice (A) and EMP and Alda-1 combinedly treated ALDH2*2 mutant diabetic mice (E + A). Data are presented as mean ± standard error of the mean (SEM). n = 8. * *p* < 0.05 and ** *p* < 0.01 refer to the difference between individual groups as shown.

We found that vehicle-treated ALDH2*2 mice with diabetes-associated HFpEF ran shorter distances (Figure 3A) and duration (Figure 3B) until exhaustion compared to non-diabetic control ALDH2*2 mice. Treatment with EMP or Alda-1 and their combination improved the running distance and duration in mice with diabetes-associated HFpEF compared to vehicle treatment (Figure 3A,B). However, the combination of EMP + Alda-1 improved the running capacity of the mice with diabetes-associated HFpEF better than EMP alone, as indicated by distance and duration (Figure 3A,B). The running distance is positively correlated with % cardiac output (CO) (Figure 3C) and % EF (Figure 3D).

### 2.5. Effects of EMP, Alda-1 and EMP + Alda-1 on ALDH2 Activity 

**Figure 4:**
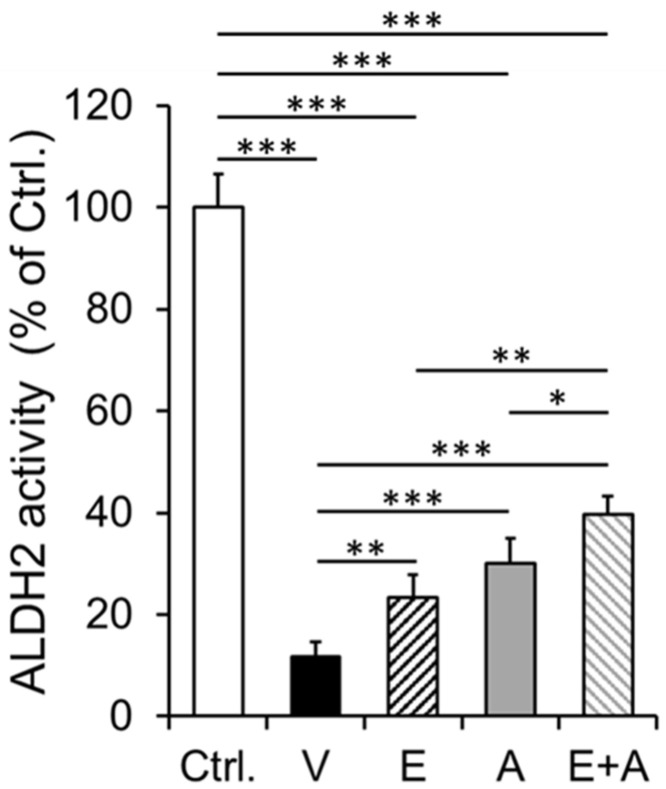
Cardiac ALDH2 activity in diabetic ALDH2*2 mutant mice exhibiting HFpEF with vehicle, EMP, Alda-1 and EMP + Alda-1 treatments. Cardiac ALDH2 activity data of non-diabetic control ALDH2*2 mutant mice (Ctrl.), vehicle-treated ALDH2*2 mutant diabetic mice (V), EMP-treated ALDH2*2 mutant diabetic mice (E), Alda-1 treated ALDH2*2 mutant diabetic mice (A) and EMP and Alda-1 combinedly treated ALDH2*2 mutant diabetic mice (E + A) were shown. Data are presented as mean ± standard error of the mean (SEM). * *p* < 0.05, ** *p* < 0.01 and *** *p* < 0.001 refer to the difference between individual groups as shown. n = 4.

We found that the myocardial ALDH2 activity was significantly reduced in vehicle- treated ALDH2*2 mice with diabetes-associated HFpEF relative to non-diabetic control mice (Figure 4). EMP, Alda-1 and EMP + Alda-1 treatments improved ALDH2 activity significantly (Figure 4). However, the combination of EMP + Alda-1 potentiated the activation of cardiac ALDH2 activity significantly compared to EMP or Alda-1 alone (Figure 4). The increase in ALDH2 activity with EMP + Alda-1 treatment was much higher compared to EMP, than Alda-1 (*p* < 0.05 vs. *p* < 0.01). However, in the individual treatments, Alda-1 increased the ALDH2 activity and was more pronounced than EMP (*p* < 0.01 vs. *p* < 0.001) (Figure 4).

### 2.6. Effects of EMP, Alda-1 and EMP + Alda-1 on 4HNE Protein Adducts 

**Figure 5:**
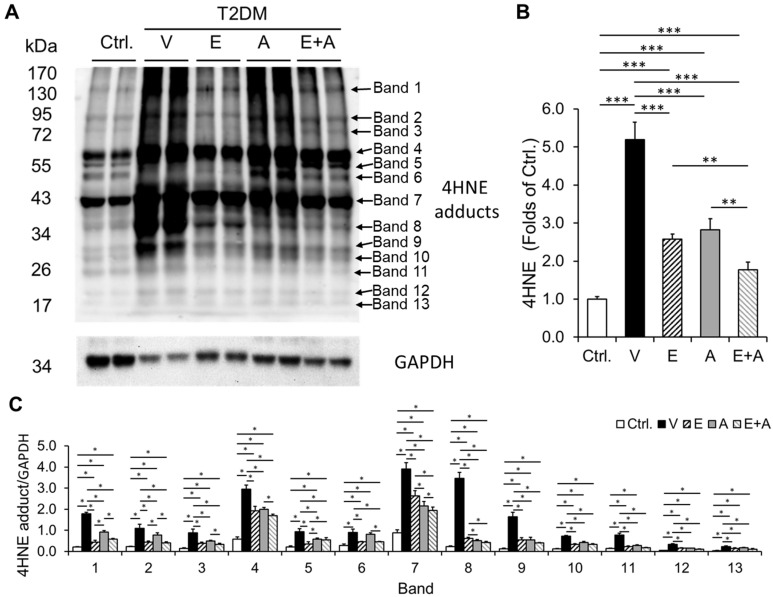
Immunoblotting band images of 4HNE protein adducts and their quantitation data in diabetic ALDH2*2 mutant mice exhibiting HFpEF with vehicle, EMP, Alda-1 and EMP + Alda-1 treatments. Western blot gel images of 4HNE protein adducts and the loading control, GAPDH of cardiac tissue samples from Ctrl, V, E, A and E + A groups (**A**), their densitometric quantification data (**B**) and the individual 4HNE protein adduct bands (**C**) were shown. Data are presented as mean ± standard error of the mean (SEM). n = 4. ** *p* < 0.01 and *** *p* < 0.001 refer to the difference between individual groups as shown in (**B**). * *p* < 0.05 refer to the difference between individual groups as shown in (**C**).

Cardiac levels of 4HNE protein adducts were higher in vehicle-treated ALDH2*2 diabetic mice with HFpEF relative to non-diabetic control mice (Figure 5A–C). These increased cardiac 4HNE levels were attenuated by EMP or Alda-1 treatment (Figure 5A–C). Finally, EMP + Alda-1 combination augmented the decrease in 4HNE protein adduct levels compared to individual treatments with EMP or Alda-1 (Figure 5A–C). 

### 2.7. Effects of EMP, Alda-1 and EMP + Alda-1 on LKB1-AMPK Signaling

**Figure 6:**
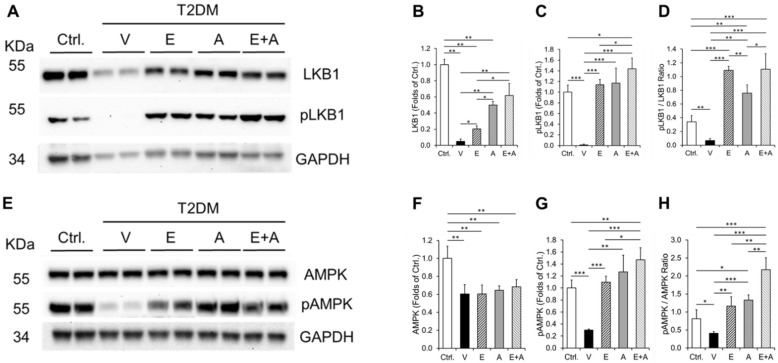
Levels of LKB1, phospho LKB1, phospho LKB1/LKB1, AMPK, phospho AMPK and phospho AMPK/AMPK in diabetic ALDH2*2 mutant mice exhibiting HFpEF with vehicle, EMP, Alda-1 and EMP + Alda-1 treatments. Representative Western blot images of LKB1, phospho LKB1 (pLKB1 i.e., Ser428) and loading control GAPDH (**A**) and the respective densitometric quantification data of LKB1 (**B**), pLKB1 (**C**), pLKB1/LKB1 (**D**). Representative Western blot images of AMPK, phospho AMPK (pAMPK i.e., Thr172) and loading control GAPDH (**E**) and the respective densitometric quantification data of AMPK (**F**), pAMPK (**G**) and pAMPK/AMPK (**H**) were shown. Data are presented as mean ± standard error of the mean (SEM). n = 4. * *p* < 0.05, ** *p* < 0.01 and *** *p* < 0.001 refer to the difference between individual groups as shown in (**B**–**D**) to (**F**–**G**).

We found a significant decrease in the LKB1 and phospho LKB1 (Ser428) levels with vehicle treatment, which was increased with EMP or Alda-1 (Figure 6A–D). However, this LKB1 increase was further significantly augmented with EMP + Alda-1 treatment (Figure 6A,B). The ratio of phospho LKB1 and LKB1 was significantly reduced in vehicle treatment compared to the controls (Figure 6A,D). Treatment with EMP, Alda-1 and EMP + Alda-1 increased the ratio of phospho LKB1 and LKB1 (Figure 6A,D). We found a significant decrease in AMPK levels with vehicle treatment compared to control (Figure 6E,F). This decrease was not significantly changed with EMP, Alda-1 and EMP + Alda-1 (Figure 6E,F). The phospho AMPK (Thr 172) levels with vehicle treatment (Figure 6E,G) was significantly reduced compared to controls mice. EMP or Alda-1 as well as a combination of EMP + Alda-1 increased phospho AMPK levels compared to vehicle treatment (Figure 6E,G). In that, EMP + Alda-1 treatment augmented the increase in the phospho AMPK levels significantly even when compared to EMP alone treatment (Figure 6E,G). The ratio of phospho AMPK/AMPK was significantly reduced in vehicle treatment compared to the controls (Figure 6E,H). The ratio of phospho AMPK/AMPK was significantly increased with EMP, Alda-1 and EMP + Alda-1 treatments (Figure 6E,H). EMP + Alda-1 treatment mediated increase was significantly higher compared to EMP or Alda-1 (Figure 6E,H). 

### 2.8. Effects of EMP, Alda-1 and EMP + Alda-1 on 4HNE Adduction on LKB1

**Figure 7:**
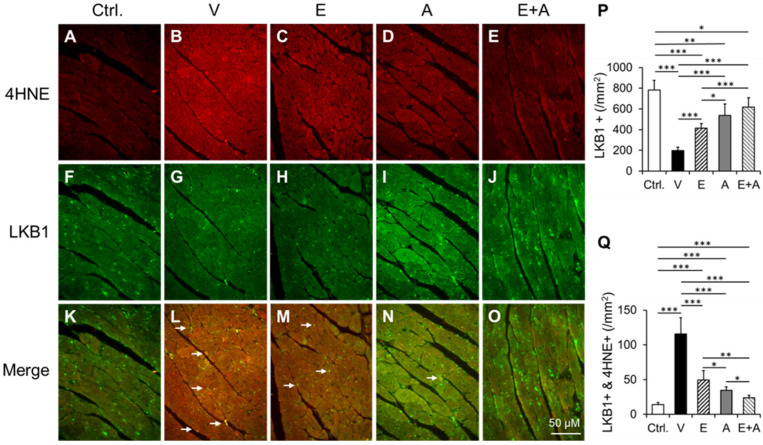
Co-immunostaining of LKB1 and 4HNE protein adducts in the cardiac sections. Representative micrographs of cardiac sections with immunostaining of LKB1 and 4HNE adducts and their merging from non-diabetic ALDH2*2 control mice (Ctrl) (**A**,**F**,**K**), ALDH2*2 diabetic mice exhibiting HFpEF with vehicle (V) (**B**,**G**,**L**), (E) (**C**,**H**,**M**), (A) (**D**,**I**,**N**) and E + A (**E**,**J**,**O**) treatments. The white arrows show co-immunopositivity of LKB1 and 4HNE protein adducts. Quantification data of LKB1+ (**P**) and co-immunostaining of 4HNE with LKB1 (**Q**) was shown. Data are presented as mean ± standard error of the mean (SEM). n = 4. * *p* < 0.05, ** *p* < 0.01 and *** *p* < 0.001 refer to the difference between individual groups as shown in (**P**,**Q**).

We found a significant increase in colocalization of LKB1 and 4HNE in the hearts of vehicle-treated ALDH2*2 mice with diabetes-associated HFpEF compared to the non-diabetic controls, implicating increased 4HNE adduction on LKB1 (Figure 7A,B,F,G,K,L,P,Q). EMP or Alda-1 significantly decreased 4HNE adduction on LKB1 compared to vehicle treatment (Figure 7C,D,H,I,M,N,P,Q). The combination of EMP + Alda-1 treatment decreased 4HNE adduction on LKB1 compared to vehicle as well as EMP or Alda-1 (Figure 7B–Q). It appears that most of this colocalization occurs at the coronary endothelial cells and some in blood cells in the blood vessels (Figure 7K–O).

### 2.9. Effects of EMP, Alda-1 and EMP + Alda-1 on 8-OHdG

**Figure 8:**
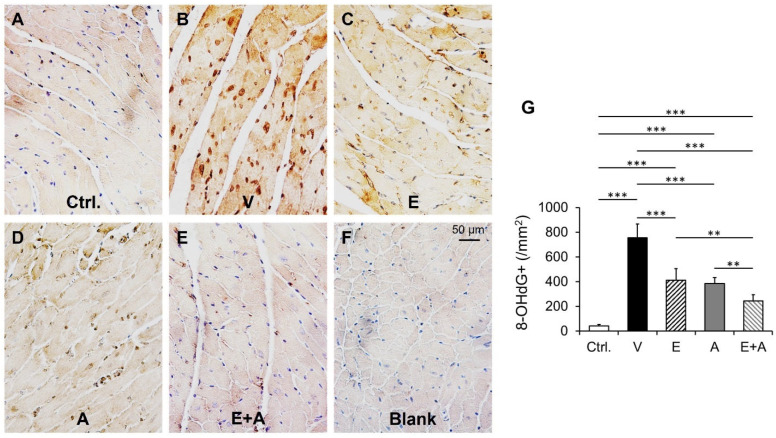
Changes in cardiac 8OHdG+ in diabetic ALDH2*2 mutant mice exhibiting HFpEF with vehicle, EMP, Alda-1 and EMP + Alda-1 treatments. Representative micrographs of 8OHdG immunostained sections from control (**A**), vehicle-treated ALDH2*2 mutant diabetic mice (**B**), EMP-treated ALDH2*2 mutant diabetic mice (**C**), Alda-1 treated ALDH2*2 mutant diabetic mice (**D**) and EMP and Alda-1 combinedly treated ALDH2*2 mutant diabetic mice (**E**). Blank (negative control) was shown in (**F**). The quantification of 8OHdG+ in cardiac tissue was shown (**G**). Data are presented as mean ± standard error of the mean (SEM). n = 4. ** *p* < 0.01 and *** *p* < 0.001 refer to the difference between individual groups as shown.

We found a significant increase in myocardial 8-OHdG, an index of oxidative DNA damage, in vehicle-treated ALDH2*2 mice with diabetes-associated HFpEF in relation to control (Figure 8A,B,G). EMP or Alda-1 significantly reduces myocardial 8-OHdG (Figure 8A–D,G). EMP + Alda-1 induced decrease in myocardial 8-OHdG was significantly higher than EMP or Alda-1 (Figure 8A–E,G). The negative control in Figure 8F explains the true immunopositivity in Figure 8A–E.

### 2.10. Effects of EMP, Alda-1 and EMP + Alda-1 on Cardiomyocyte Hypertrophy 

**Figure 9:**
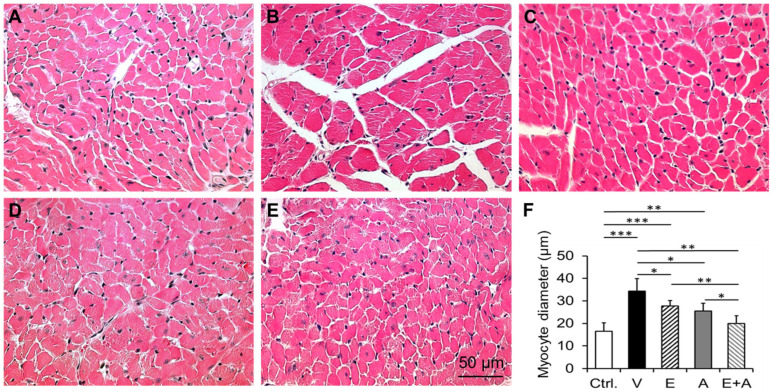
Changes in cardiomyocyte hypertrophy in diabetic ALDH2*2 mutant mice exhibiting HFpEF with vehicle, EMP, Alda-1 and EMP + Alda-1 treatments. Representative micrographs of hematoxylin-eosin (H-E) stained sections from control (**A**), vehicle-treated ALDH2*2 mutant diabetic mice (**B**), EMP-treated ALDH2*2 mutant diabetic mice (**C**), Alda-1 treated ALDH2*2 mutant diabetic mice (**D**) and EMP and Alda-1 combinedly treated ALDH2*2 mutant diabetic mice (**E**). The quantification of cardiomyocyte hypertrophy was shown (**F**). Data are presented as mean ± standard error of the mean (SEM). We have randomly picked n = 4 from the 8 mice. * *p* < 0.05, ** *p* < 0.01 and *** *p* < 0.001 refer to the difference between individual groups as shown.

We found a significant increase in cardiomyocyte hypertrophy in vehicle-treated ALDH2*2 mice with HFpEF after type 2 DM in relation to control mice (Figure 9A,B,F). EMP or Alda-1 significantly reduces cardiomyocyte hypertrophy (Figure 9A,D,F). EMP + Alda-1 induced a decrease in cardiomyocyte hypertrophy and was significantly higher than EMP or Alda-1 (Figure 9A–F). This decrease by EMP + Alda-1 was significantly higher in EMP (*p* < 0.01) than Alda-1 (*p* < 0.05).

### 2.11. Effects of EMP, Alda-1 and EMP + Alda-1 on Myocardial Fibrosis 

**Figure 10:**
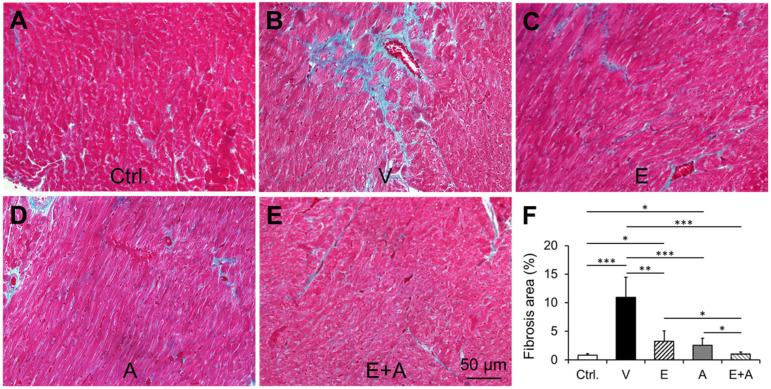
Changes in myocardial fibrosis in diabetic ALDH2*2 mutant mice exhibiting HFpEF with vehicle, EMP, Alda-1 and EMP + Alda-1 treatments. Representative micrographs of Mason Trichrome stained sections from control (**A**), vehicle-treated ALDH2*2 mutant diabetic mice (**B**), EMP-treated ALDH2*2 mutant diabetic mice (**C**), Alda-1 treated ALDH2*2 mutant diabetic mice (**D**) and EMP and Alda-1 combinedly treated ALDH2*2 mutant diabetic mice (**E**). The quantification of myocardial fibrosis was shown (**F**). Data are presented as mean ± standard error of the mean (SEM). n = 4. * *p* < 0.05, ** *p* < 0.01 and *** *p* < 0.001 refer to the difference between individual groups as shown.

We found a significant increase in myocardial fibrosis in vehicle-treated ALDH2*2 mice with diabetes-associated HFpEF in relation to control (Figure 10A,B,F). EMP or Alda-1 significantly reduces myocardial fibrosis (Figure 10A–D,F). EMP + Alda-1 induced decrease in myocardial fibrosis was significantly higher than EMP or Alda-1 (Figure 10A–E,F). 

## 3. Discussion

Combined treatment of EMP + Alda-1 ameliorated the hallmarks of diabetes-associated HFpEF, such as cardiac diastolic function and exercise capacity as well as decreased cardiomyocyte hypertrophy and myocardial fibrosis, more than the individual treatment with EMP in ALDH2*2 mice. This effect was rendered by decreasing cardiac 4HNE protein adducts and 8OHdG levels significantly with a notable improvement in the LKB1-AMPK signaling along with attenuating 4HNE adduction on LKB1. In the present study, Alda-1 treatment alone improved the cardiac function and exercise tolerance without decreasing hyperglycemia but increasing 4HNE detoxification in ALDH2*2 mutant diabetic mice with HFpEF. Thus, 4HNE may be critical in contributing to the HFpEF pathophysiology. Alda-1 exerted greater improvements in cardiac function, exercise tolerance and increasing LKB1 level, phospho LKB1 (ser 428) and their ratio compared to EMP. From our study, we can point out that Alda-1 increased the metabolism of 4HNE, thus protecting the myocardium from HFpEF. However, the combination treatment of EMP + Alda-1 showed greater benefits. 

As we chose diabetes-associated HFpEF as a model for our study, such comorbidities-specific strategies in HFpEF will be beneficial, and should also fit with the emerging concept of precision medicine. Among the global diabetic population, roughly 40% of the diabetics are Asians [17], equating to ~150 million people with a high mortality rate. Several studies have indicated that Asians develop diabetes at a younger age compared to their Caucasian counterparts [17]. East Asians with the ALDH2*2 mutation have a higher incidence of cardiovascular and metabolic diseases [18], such as diabetes [19], diabetes-induced neuropathy and vasculopathy [20] and diabetes-induced diastolic dysfunction [21]. Our ALDH2*2 E487K knock-in mutant mice mimic East Asians with the ALDH2*2 E487K mutation [22,23] and we demonstrated that this ALDH2*2 E487K knock-in mutant mice with HFD-fed type-2 diabetes recapitulated major characteristics of HFpEF [6]. We found increased diastolic dysfunction with preserved systolic function and poor exercise intolerance in these ALDH2*2 mice mimicking HFpEF features. The evidence to substantiate this were decreased relaxation rate [the details of its calculation is provided in our previous report [6]] along with preserved EF (>60%) and FS (>45%). In fact, in a recent clinical study, the ALDH2*2 mutant variant is associated with an increased risk of HFpEF in patients with hypertension, diabetes and coronary heart disease [14]. Most importantly, diabetes was shown to decrease ALDH2 activity in non-ALDH2*2 mutant animals i.e., WT animals as well [24]. Thus, our research findings will be applicable to all patients with metabolic stress, more so for East Asians with the ALDH2*2 mutation. We have recently shown that ALDH2*2 mutant diabetic hearts have reduced coronary endothelial density and Alda-1 can reduce the coronary endothelial cell damage in the myocardium [25], since HFpEF originates from systemic oxidative stress, inflammation, and endothelial cell dysfunction at the cardiac tissue levels. We presume Alda-1 prevented 4HNE-mediated inflammation and thereby ameliorated HFpEF in the current model. To specifically understand the role of 4HNE in the coronary endothelial cell damage, we employed mouse coronary endothelial cells in our recent studies [11,12]. In fact, this study also displays that increased 4HNE adduction in LKB1 was higher in coronary endothelial cells (Figure 7). 

Similar to Alda-1, EMP alone improved cardiac function and exercise tolerance in the mice with HFpEF by decreasing hyperglycemia-mediated 4HNE adducts. Notably, EMP is gaining importance in T2DM patients as its benefits extend beyond its glucose-lowering action [26,27,28]. Specifically, its effect, which led to improvements in heart failure patients and cardiovascular death, is striking in the landmark randomized clinical trial (RCT) EMPA-REG OUTCOME (Empagliflozin Cardiovascular Outcome Event Trial in Type 2 Diabetes Mellitus Patients) [29]. A meta-analysis of RCTs has also confirmed the benefits of EMP [30]. Gupte et al. [26] summarized clinical, preclinical and meta-analysis of SGLT2 inhibitors mediated cardiac benefits. Ongoing RCTs are planned to study the effects of EMP in HFpEF [31,32]. For instance, SUGAR-DM-HF (Studies of Empagliflozin and Its Cardiovascular, Renal and Metabolic Effects) RCT will check for the effects of empagliflozin on specific pathways in cardiac and renal effects in patients with type 2 diabetes (or pre-diabetes) and HF. In a study using explanted cardiac papillary muscle from murine (diabetic and non-diabetic) and human HF subjects, EMP directly reduced diastolic dysfunction via improving myofilament function with enhanced myofilament phosphorylation [33]. 

As roughly 130 million diabetics live in the Western Pacific region, with a significant proportion in East Asia including China, Philippines, Japan and Korea [17], a sub analysis of EMP treatment in the Asian population within EMPA-REG OUTCOME was performed; it revealed that EMP was beneficial to Asians as well [34], which underscores our interest to focus on the ALDH2*2 mutation and increases the propensity to develop diabetic cardiac dysfunction [21] as well as HFpEF in East Asians [14]. There are few diabetic animal studies where the beneficial cardiovascular effects of EMP were shown [26,35,36], including our previous study in which EMP was shown to improve cardiac function and exercise tolerance in the same animal model by diminishing hyperglycemia-mediated 4HNE adducts [15]. 

The highlight of this study is the evaluation of the EMP + Alda-1 combination mediated pharmacological effects on diabetes-associated HFpEF. The addition of Alda-1 to EMP improved cardiac diastolic dysfunction before exercise stress. After the exercise stress, the combination treatment showed improvements in both diastolic and systolic function besides increasing heart rate. At the cell signaling level, the combination treatment was better in increasing ALDH2 activity, reducing 4HNE protein adducts level and improving the LKB1-AMPK signaling pathway, i.e., the increase in the Ser428 pLKB1 and Thr172 pAMPK, and decreasing 4HNE adduction on LKB1. Earlier studies implicated that cardiac-specific haplodeficiency of LKB1 in mice fed with HFD and a high-sucrose diet causes deleterious effects at multiple levels [37], including: (1) cardiac functional level—diastolic dysfunction and systolic failure; (2) cellular level—apoptosis, hypertrophy and fibrosis; (3) organelle level—mitochondrial dysfunction as well as (4) signaling level—decrease in AMPK phosphorylation. Pertaining to our line of thinking, 4HNE adduction on essential signaling proteins afflicts cardiac tissue and causes abnormal function [37]. For instance, in spontaneously hypertensive rats, 4HNE adduction on LKB1 in hypertrophic myocardium was shown in reducing AMPK phosphorylation [38]. In that report, 4HNE-mediated inhibition of the LKB1/AMPK signaling was established as critical signaling in the hypertrophic process [38]. Furthermore, at the molecular level, modification at Lys-97 by 4HNE alone can inhibit LKB1 [39]. Even though we did not perform such molecular studies in our current study, our interpretation of the results suggest that decreasing 4HNE protein adduct levels by two different pharmacological approaches protects LKB1 from 4HNE adduction and thus improving its activity as evident from increased phosphorylation of LKB1 at Ser428 and then its substrate, AMPK at Thr172. Therefore, we propose that efficient removal of cardiac tissue at the 4HNE level by both augmenting ALDH2 activation mediated 4HNE detoxification, along with decreasing hyperglycemia-mediated 4HNE production, will be beneficial in ameliorating diabetes-associated HFpEF, which can be tested in other HFpEF models where oxidative stress ensues. Subcutaneously injected Elamipretide (MTP-131), a novel mitochondria-targeting peptide, into heart failure dogs for 3 months significantly decreased cardiac 4HNE, increased LV ejection fraction and improved mitochondrial respiration in treated dogs [40]. Even though it may be just concurrence however, further studies on large animals such as dogs and pigs will solidify that decreasing 4HNE will be beneficial in ameliorating HFpEF. In a type-1 diabetic model we have also shown that decreasing 4HNE adduction reduced cardiac damage [5]. 

In conclusion, 4HNE-protein adduction-mediated cell signaling aberrations are critical in HFpEF. Thus, targeting 4HNE removal from the organ systems can serve as a key strategy to alleviate HFpEF.

## 4. Materials and Methods

### 4.1. High-Fat Diet (HFD)-Fed Type-2 Diabetes Mellitus in ALDH2*2 Mutant Heterozygous Mice 

ALDH2*2 knock-in mutant male heterozygous mice (with C57BL/6J background) with 3–4 months of age were fed an HFD (60% of calories from fat, D12492, Research Diets) as we published earlier [6]. Normal chow-fed ALDH2*2 mice were served as controls. In the HFD-fed group, the mice with sustained elevated fasting blood glucose levels above 250 mg/dL were deemed as diabetic mice and selected for further treatments. ALDH2*2 mice were inbred in-house and genotyped by Transnetyx Inc., Cordova, TN, USA. The animal protocol was approved by the Henry Ford Health System and Wayne State University Institutional Animal Care and Use Committee. We confirm that all experiments were performed in accordance with IACUC guidelines and regulations. All the animal experiments followed the ethical guidelines of ARRIVE.

### 4.2. Intraperitoneal Glucose Tolerance Test (IPGTT)

The IPGTT was performed in ALDH2*2 knock-in mutant male heterozygous mice from control and diabetic groups as explained elsewhere [4]. After fasting for 6 h, the mice were injected with 2 g/kg D-glucose. Then, blood glucose levels were measured at 0, 30, 60, 90 and 120 min after D-glucose injection, using a glucometer. 

### 4.3. Pharmacological Treatment Protocols

At the end of 6 months, we divided the diabetic ALDH2*2 heterozygous mice randomly into 4 groups with n = 8 animals for each group: DMSO as vehicle (Veh); an SGLT2 inhibitor, EMP (3 mg/kg/d) to reduce hyperglycemia; an ALDH2 activator, Alda-1 (10 mg/kg/d) to enhance ALDH2 activity (Alda-1); as well as a combination of EMP + Alda-1. These agents were administered via using Alzet Mini-osmotic pumps (Model # 2004; 0.25 μL per hour, 28 days) by implanting subcutaneously (we changed the pumps after 28 days for one more time to administer for 56 days (i.e., 8 weeks). We selected the dose of EMP from previously published studies from us [15] and others [41,42,43]. Similarly, Alda-1 dose was selected from previous studies [44,45,46]. After 8 weeks of treatments, cardiac function was assessed by conscious echocardiography post-exercise stress.

### 4.4. Cardiac Function Assessment by Echocardiography in Conscious Mice

To avoid the effects of anesthesia, we assessed left ventricular dimension and function in conscious mice using an echocardiograph equipped with a 15-MHz linear transducer (Acuson c256, Malvern, PA, USA) as we described previously [4,6,47]. 

### 4.5. Acute Progressive Maximal Exercise Test (Exhaustion Test)

The non-diabetic control ALDH2*2 mice and diabetic ALDH2*2 mice treated with vehicle, EMP, Alda-1 and EMP + Alda-1 combination were subjected to the exercise exhaustion test as described earlier [6]. We calculated running distance of the mice from the recording. 

### 4.6. Post-Exercise Echocardiography

Immediately after finishing the exercise exhaustion test, we performed conscious echocardiography again on the mice to record their functional changes. We calculated heart rate, fractional shortening (% FS) and % ejection fraction (% EF) for systolic functional parameters and, relaxation rate (RR) and left atrial (LA) area for diastolic dysfunction. 

### 4.7. Histopathology

The middle portions of the cardiac tissue were fixed with 10% formalin in PBS, embedded in paraffin as blocks, and several transverse sections were cut for histopathological studies. 

### 4.8. Measurement of Cardiomyocyte Hypertrophy

The myocardial sections were stained with hematoxylin-eosin (H-E). H-E-stained cardiac sections were used to measure cardiomyocyte diameter to determine hypertrophy as we reported earlier [4,5]. We randomly picked 4 samples from each group to measure cardiomyocyte diameter.

### 4.9. Measurement of Cardiac Fibrosis

The myocardial sections were stained with Mason’s Trichrome. The red color indicates the deposition of collagen and this area was measured using the MicroSuite Basic software (Olympus America, Center Valley, PA, USA). The percent (%) area of fibrosis was quantified from each tissue section as previously described [5,25]. We randomly picked 4 samples from each group to measure cardiac fibrosis. 

### 4.10. ALDH2 Activity Assay

The cardiac ALDH2 activity was measured as we described in previous reports [4,12]. In brief, the protein samples were allowed to react with 50 mM sodium pyrophosphate as a buffer, 2.5 mM NAD+ as a cofactor and 10 mM acetaldehyde as substrate. The reductive reaction of NAD+ to NADH at λ340 nm wavelength at 37 °C temperature was measured as ALDH2 activity. We randomly picked 4 samples from each group to measure ALDH2 activity.

### 4.11. Western Immunoblotting

The Western blot was performed as described earlier (6, 13). In brief, protein samples from cardiac tissue were separated on SDS-polyacrylamide gels by electrophoresis and transferred to immobilon-P membranes (Millipore, Billerica, MA, USA). The changes in the protein levels in cardiac tissue samples were determined using antibodies of anti-4HNE-Cys/His/Lys rabbit antibody (Millipore, Billerica, MA, USA), anti-liver kinase B1 (LKB1) antibody (Cell Signaling Technology, Danvers, MA, USA), anti-phospho LKB1 (Ser428) antibody (Cell Signaling Technology, Danvers, MA, USA), AMP-activated protein kinase (AMPK) and anti-phospho AMP-activated protein kinase (Thr 172) antibody (Cell Signaling Technology), along with anti-GAPDH antibody (Santacruz) that was used as a housekeeping marker and loading control. The horseradish peroxidase (HRP)-coupled respective secondary antibodies were added to form complexes with primary antibodies which were then visualized by using chemiluminescence detection reagents. We randomly picked 4 samples from each group to do Western immunoblotting.

### 4.12. Immunohistochemistry Staining of 8-Hydroxy-2′-Deoxyguanosine (8OHdG)

Formalin-fixed, paraffin-embedded cardiac biventricular tissue sections were used for immunohistochemical staining. After deparaffinization and hydration, the slides were washed in Tris-buffered saline (TBS; 10 mmol/L Tris–HCl, 0.85% NaCl, pH 7.5) containing 0.1% bovine serum albumin (BSA). Endogenous peroxidase activity was quenched by incubating the slides in 0.6% H_2_O_2_/methanol. A pressure cooker method was used to retrieve the antigen. In the following steps, reagents from an immunoperoxidase staining kit (Millipore, Billerica, MA, USA) were used as directed. A solution from the kit was used to block non-specific reactions. After overnight incubation with mouse 8OHdG antibody (Abcam, Cambridge, MA, USA) at a concentration of 1:200 and 4 °C, the slides were washed in TBS. Secondary antibody solution and streptavidin peroxidase solution were added and incubated at room temperature. Immunostaining was visualized with chromogen, diaminobenzidine tetrahydrochloride (DAB). After overnight incubation with mouse 8OHdG antibody (Abcam, Cambridge, MA, USA) at a concentration of 1:200 at 4 °C, the slides were washed in TBS. We counted the brown color spots as 8OHdG positivity in the cardiac sections from various groups using light microscope (Olympus America, Center Valley, PA, USA) and plotted them as a graph as we reported earlier [24,25]. We randomly picked 4 samples from each group to perform immunostaining of 8OHdG.

### 4.13. Immunofluorescence Staining of LKB1 and 4HNE Adducts

Formalin-fixed, paraffin-embedded cardiac biventricular tissue sections were used for immunostaining as we explained elsewhere [24]. After deparaffinization and hydration, the slides were washed in Tris-buffered saline (TBS; 10 mmol/L Tris–HCl, 0.85% NaCl, pH 7.5) containing 0.1% bovine serum albumin (BSA). A pressure cooker method was used to retrieve the antigen. In the following steps, reagents from an immunoperoxidase staining kit (Millipore) were used as directed. A solution from the kit was used to block non-specific reactions. The following antibodies were used for co-immunostaining at a concentration of 1:100 and 4 °C for overnight incubation: anti-LKB1 mouse monoclonal antibody (Thermofischer Scientific Inc., Waltham, MA, USA) and anti-4HNE-Cys/His/Lys rabbit polyclonal antibody (Millipore Sigma, Burlington, MA, USA). The secondary antibodies were conjugated with FITC and rhodamine (Thermofischer Scientific Inc. Waltham, MA, USA) at a concentration of 1:500 at room temperature for 1 h. Immunofluorescence positive staining was analyzed using an Olympus microscope and an image analyzer. We randomly picked 4 samples from each group to perform co-immunostaining of LKB1 and 4HNE protein adducts. 

### 4.14. Statistical Analysis

Data are presented as mean ± standard error of the mean (S.E.M). We used One-Way ANOVA for group comparisons and the post-hoc analysis was performed using Student *t*-test. The differences between before and after exercise stress with individual groups were analyzed by using the paired Student *t*-test. We used Microsoft Excel 2013 for all statistical analysis. 

## 5. Perspectives

This study shows that combined treatment with EMP, a SGLT2 inhibitor that lowers hyperglycemia and Alda-1, an ALDH2 activator, improved exercise tolerance and diastolic function better than EMP alone in a mouse model of HFpEF associated with diabetes. This was mediated by reduced cardiac 4HNE protein adduct levels due to decreased 4HNE production via EMP and augmented 4HNE detoxification by Alda-1.

## Data Availability

Not Applicable.
